# Population pharmacokinetics of gentamicin in acute lymphoblastic leukemia pediatric patients compared to non-oncology patients

**DOI:** 10.1016/j.jsps.2024.102060

**Published:** 2024-04-01

**Authors:** Hisham S. Abou-Auda, Fatimah Alotaibi, Sary Alsanea, Abdulrahman Alwhaibi, Mohammed M. Almutairi, Ziyad Alrabiah, Abdullah Alsultan, Majed Al Jeraisy

**Affiliations:** a Department of Clinical Pharmacy, College of Pharmacy, King Saud University, Riyadh, Saudi Arabia; b King Abdullah Specialist Children's Hospital, Ministry of National Guard, Saudi Arabia; c Department of Pharmacology and Toxicology, College of Pharmacy, King Saud University, Riyadh, Saudi Arabia; d King Abdullah International Medical Research Center, Saudi Arabia; e King Saud Bin Abdulaziz University for Health Sciences, Saudi Arabia

**Keywords:** Acute Lymphoblastic Leukemia (ALL), Gentamicin, Pediatric, Pharmacokinetics (PK), Therapeutic Drug Monitoring (TDM)

## Abstract

Understanding the pharmacokinetics of gentamicin is essential in special populations, such as pediatric patients with acute lymphoblastic leukemia (ALL), in light of previous studies indicating that ALL patients have a lower volume of distribution than non-ALL patients. Furthermore, validation of such results is needed to ensure their clinical application. Accordingly, this single-center, retrospective, cross-sectional study compares the pharmacokinetic parameters of volume of distribution and clearance (Cl) of gentamicin between ALL and non-ALL patients. Inclusion criteria were pediatric patients aged between 1 and 14 years with or without ALL and receiving intravenous gentamicin for treatment courses > 72 h. Patients’ characteristics, such as age, sex, height, serum albumin, diagnosis, serum creatinine (Scr) concentration, dosing, and pharmacokinetic information, including peak and trough concentrations, were retrieved. The study scrutinized a total of 115 pediatric patients, comprising toddlers (15.7 %), children (76.5 %), and adolescents (7.8 %). All patients received gentamicin every 8 h, with an average dose of 2.50 (0.64) mg/kg. Patients were divided into two groups based on disease state, with 45.2 % (n = 52) in the non-ALL group and 54.8 % (n = 63) in the ALL group. Both groups had similar characteristics in terms of gender, weight, body surface area, and dose. The only significant covariates identified were weight and creatinine clearance (Cl_cr_) for volume of distribution (V_d_). A significant difference was found in Scr, Cl_cr_, and blood urea nitrogen (BUN); however, no significant difference between ALL and non-ALL patients emerged in the volume of distribution or Cl. In conclusion, the study findings indicate that dosing requirements were similar between the two groups. Further prospective studies with larger sample sizes are warranted.

## 1 Introduction

Gentamicin is one of the most commonly used aminoglycoside (AG) antibiotics in hospitalized children and is specifically applied for gram-negative bacterial infections (Llanos-Paez et al., 2020). Notably, AGs feature a narrow therapeutic window and high inter- and intra-patient variability (Dettli, 1974, Zaske et al., 1980). Therefore, therapeutic drug monitoring (TDM) is warranted to maintain serum concentrations (peak and trough) within the desired therapeutic range. Due to gentamicin’s property of concentration-dependent killing activity, the peak concentration (C_max_) over minimum inhibitory concentration (MIC) ratio must be above 10 for a better clinical effect (Kashuba et al., 1999, Mingeot-Leclercq and Tulkens, 1999, Best et al., 2011, Bland et al., 2018).

A hydrophilic molecule, gentamicin is mostly eliminated by renal excretion (∼90 %) through glomerular filtration (He et al., 2022a). In healthy individuals, the clearance (Cl) of gentamicin is approximately 90 ml/min, while the apparent volume of distribution (V_d_) is 0.25–0.5 L/kg, and the elimination half-life is 2–3 h (Hansen et al., 2001, He et al., 2022a, He et al., 2022b). In acute lymphoblastic leukemia (ALL), the drug’s pharmacokinetics (PK) is significantly influenced by physiological changes associated with the use of chemotherapy, such as changes in fat mass and renal function (Fleming and Capizzi, 1993, Halton et al., 1998, Orgel et al., 2021). Adult oncology study findings point to increased Cl and V_d_ (Higa and Murray, 1987, Zeitany et al., 1990, Rosario et al., 1998) or an increase in V_d_ only (Phillips et al., 1988). Similar results have been reported in oncology pediatric patients (Ho et al., 1995, Bialkowski et al., 2016). Nonetheless, one study involving oncology and non-oncology pediatric patients revealed a 15 % reduction in central V_d_ in the oncology group (Llanos-Paez et al., 2020). The observed differences in V_d_ were attributable to the considerably larger percentage of fat mass in children undergoing cancer therapy compared to non-oncology pediatric patients. In addition to reduced central V_d_, a 32 % reduction in Cl was also observed in the oncology group compared to non-oncology patients. These findings indicate that oncology patients might require lower dosages than non-oncology patients. In this light, more research is needed to understand PK variations among these patients. Accordingly, the main objective of this retrospective cross-sectional study is to compare the PK parameters, particularly V_d_ and Cl, of gentamicin between ALL and non-ALL patients and investigate whether these parameters impact gentamicin dosing in ALL patients.

## 2 Methods

### 2.1 Study design and setting

This retrospective single-center cross-sectional study was conducted at King Abdullah Specialized Children’s Hospital (KASCH). The study protocol was approved by the Institutional Review Board (approval no. NRC21R/127/04), and informed consent was waived.

The study data reflect the practice of using gentamicin in hematology/oncology patients at KASCH as empirical therapy for febrile neutropenia protocol or any suspected bacterial gram-negative infection. The usual starting dose for all pediatric patients is 2.5 mg/kg every 8 h unless the patient has compromised renal function (high serum creatinine and BUN in addition to calculating GFR) or diminished urine output. In the case of renal compromise, GFR is calculated using serum creatinine and body surface area (BSA); for GFR > 50 ml/min/1.73 m^2^, no change in the dose is made; for GFR < 50 ml/min/1.73 m^2^, the dosing guidelines for adults and children should be applied (according to the 5th ed. American College of Physicians 2007). Per hospital protocol, routine TDM is performed to determine gentamicin peak and trough levels around the third dose to ensure a steady state is reached.

### 2.2 Subjects

Included in the current study were all pediatric patients at KASCH between 1 and 14 years with or without ALL and receiving intravenous gentamicin dosage at 2.5 mg/kg/dose over 30 min every 8 h for treatment courses of more than 72 h. Critically ill pediatric patients with renal failure, liver dysfunction, or burns were excluded. Blood samples that were drawn inappropriately (late or early peak and trough levels) were also excluded. Serum samples were assayed using the latex inhibition immunoassay method (TDX, Abbott Laboratories). Serum sample measurement times were established at 30 min before the next dose (plasma trough level) and 1 h after the end of an infusion (plasma peak level).

### 2.3 Data collection

Patients’ demographic information, such as age, sex, height, weight, serum albumin, diagnosis, serum creatinine (Scr) concentration, dosing information, and gentamicin levels (peak and trough concentrations), were recorded. Creatinine clearance (Cl_cr_) was calculated using the modified Schwartz formula (Schwartz et al., 2009).

### 2.4 PK analysis

Population PK was completed using Monolix software (Version 2023R1). The data were modeled using a one-compartment system with linear elimination similar to that reported in our prior publication (Alsultan et al., 2019) since the sparse available data made it difficult to use models with > 1 compartment. In addition, PK parameters were assumed to follow a log-normal distribution. Residual variability was tested via constant, proportional, and combined error models. Individual PK parameters (empirical Bayesian estimates) were also used to assess the effect of covariates on the PK parameters of both Cl and V_d_. The tested covariates included age, sex, height, weight, serum albumin, diagnosis, Scr concentration, ALL status, and Cl_cr._ Testing for covariates was completed using linear regression with a forward approach, with the significance level set at 0.05.

The model was validated using standard goodness-of-fit plots, including observed *vs.* predicted concentration, a residual plot, and a visual predictive check.

### 2.5 Statistical analysis

Continuous data were presented as mean (SD), while categorical variables were presented as frequencies. All statistical analyses were performed using R statistical software.

## 3 Results

In all, 115 pediatric patients were included in the current study, comprising 15.7 % toddlers, 76.5 % children, and 7.8 % adolescents (Table 1). Based on the disease state, 45.2 % (n = 52) were in the non-ALL group, while 54.8 % (n = 63) were in the ALL group. Both groups had similar characteristics in terms of gender, weight, BSA, and dose. Although the differences were not significant, the ALL group tended to be younger and had a higher body mass index (BMI) compared to the non-ALL group. In contrast, significant differences were found in Scr, Cl_cr_, and BUN. Specifically, a significant increase in Cl_cr_ was observed in the non-ALL patients compared to the ALL group (164.88 *vs.* 153.7 ml/min, *p* = 0.006). However, the latter had a significantly higher Scr and BUN compared to the former (41.85 *vs.* 35.13 µmol/L, *p* = 0.005 and 3.18 *vs.* 2.38 mmol/L, *p* = 0.026, respectively). All patients received gentamicin doses every 8 h; see Table 1 for further details.

**Table 1:** Baseline demographics.

Variable	All patients (n = 115)	Acute lymphoblastic leukemia (n = 63)	Non-Acute lymphoblastic leukemia (n = 52)	*p* value
Age group [n (%)] Toddlers Children Adolescents	18 (15.7) 88 (76.5) 9 (7.8)	15 (23.8) 43 (68.3) 5 (7.9)	3 (5.8) 45 (86.5) 4 (7.7)	–
Age in years [mean (SD)]	6.1 (4.2)	5.36 (4.48)	6.88 (3.8)	0.055
Gender [number of males (%)]	63 (54.8)	34 (53.9)	29 (55.7)	–
Weight (kg)^#^	22.1 (17.1)	22.23 (20.3)	22.02 (12.2)	0.949
BMI^#^	16.9 (5.9)	17.82 (7.2)	15.67 (3.4)	0.053
BSA^#*^	0.8 (0.4)	0.77 (0.45)	0.81 (0.3)	0.617
IBW*	25.5 (13.8)	23.55 (14.5)	27.96 (12.7)	0.088
Dose (mg/dose)	54.3 (44.2)	54.80 (54.7)	53.61 (27.01)	0.885
Dose (mg/kg)	2.5 (0.64)	2.53 (0.76)	2.47 (0.44)	0.660
Frequency	every 8 h			
Cl_cr_ (ml/min) ^#*^	149 (56.5)	153.73 (62.8)	164.88 (43.5)	0.006
Scr (µmol/L)	38.8 (12.9)	41.85 (15.6)	35.13 (7.58)	0.005
BUN (mmol/L) ^#^	2.8 (1.9)	3.18 (1.9)	2.38 (1.7)	0.026

# Missing patients (1–4 patients), * Body surface area (BSA) was estimated by Mosteller equation (Mosteller, 1987) and DuBois and DuBois equation; the average of the two methods is reported (Iannessi et al., 2018). The ideal body weight (IBW) of each patient was calculated by the best guess method (Tinning and Acworth, 2007) and the New Advanced Pediatric Life Support (NAPLS) formula (Seddon et al., 2012); the calculated average from the two methods is reported. Creatinine clearance (Cl_cr_) was calculated using the modified Schwartz formula (Schwartz et al., 2009).

***PK Parameters in ALL vs. non-ALL***

**Fig. 1:**
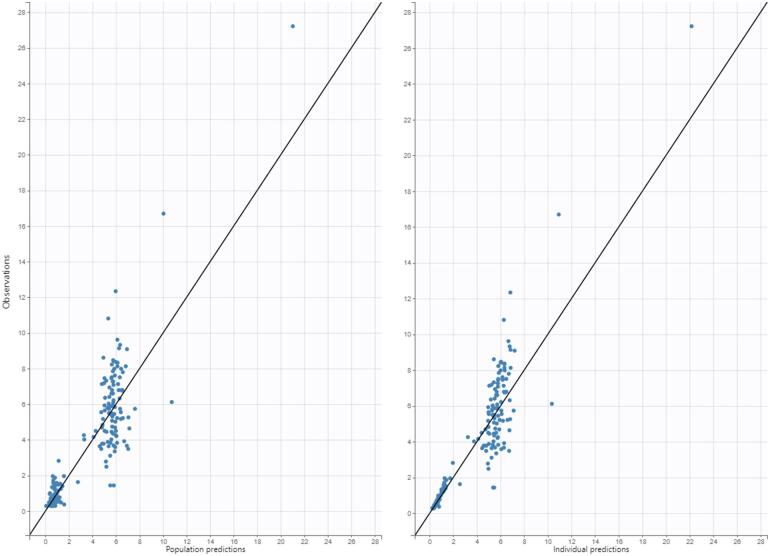
Diagnostic plot for observed concentrations *vs.* predicted concentrations: population predictions (left) and individual predictions (right).

**Fig. 2:**
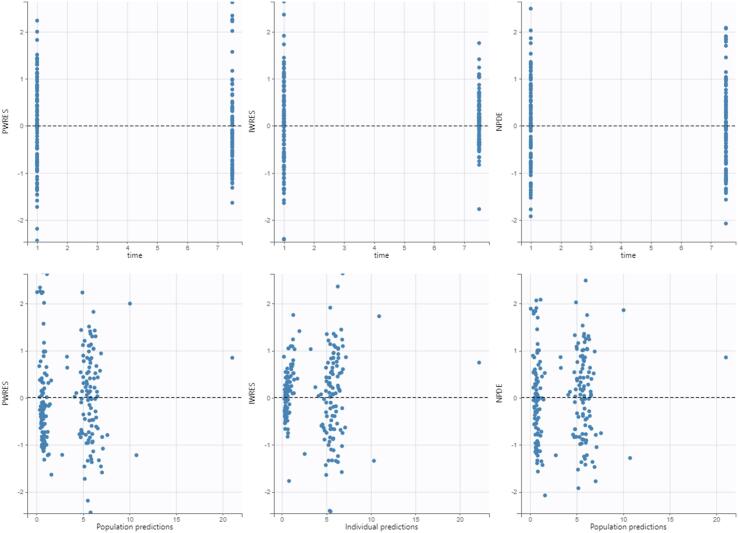
Population weighted residuals (left), individual weighed residuals plot (middle), and normalized prediction distribution error plot (right).

**Fig. 3:**
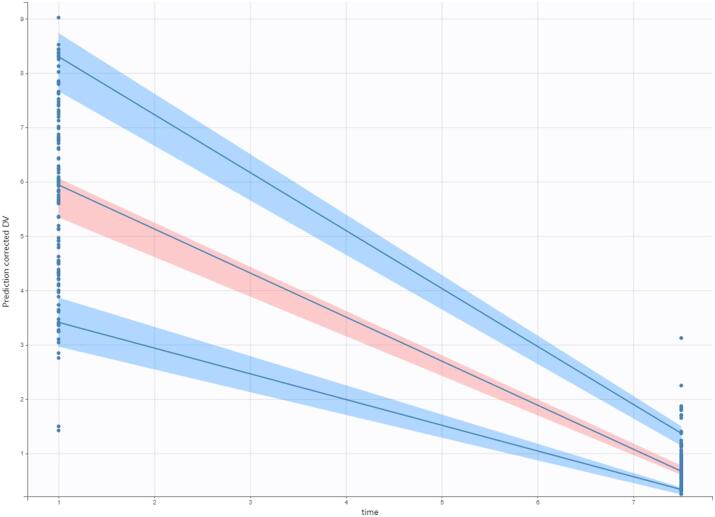
Visual predictive check plot. The solid lines represent the 10th, 50th, and 90th percentiles of observed data. The shaded regions represent the 90% confidence interval around the 10th, 50th, and 90th percentiles of simulated data. The circles are observed concentrations.

In univariate analysis for Cl, ALL status, weight, Cl_cr_, and age were significant covariates. The average Cl in ALL *vs.* non-ALL patients was 1.9 *vs.* 2.2 L/hour (*p* value > 0.05). In the multi-linear forward regression for Cl, only weight and Cl_cr_ remained significant (Table 2), while univariate analysis for V_d_ yielded only body weight as a significant covariate. The average V_d_ in ALL *vs.* non-ALL patients was 6.3 *vs.* 6.4 L (*p* value > 0.05). Goodness-of-fit plots are presented in Fig. 1, Fig. 2, Fig. 3. The effect of covariates on PK parameters was modeled using a power function as follows:

$$\mathrm{Cl}=2.13\times {\left(\frac{\mathrm{weight}}{20}\right)}^{0.75}\times {\left(\frac{\;CLcr}{120}\right)}^{0.437}$$

$$Vd=7.3\times \left(\frac{\mathrm{weight}}{20}\right)$$

The PK parameter estimates are shown in Table 3.

**Table 2:** Univariate and multivariable linear regression for Cl.

Univariate testing	*p* value	R^2^
Weight	<0.05	0.38
Age	<0.05	0.46
Oncology status	<0.05	0.07
CL_cr_	<0.05	0.35
Forward multiple linear regression		
Weight and CL_cr_	<0.05 for both	0.69

**Table 3:** Pharmacokinetic estimates for the final population pharmacokinetic model.

PK parameters	Estimate	RSE%
V (L)	7.3	3.6 %
IIV* V	14 %	28 %
Cl (L/hr)	2.3	2.7 %
IIV Cl	15.8 %	13.7 %
Residual variability B**	0.3	8.2 %

*IIV is expressed as the coefficient of variation, RSE; relative standard error.

**B is residual unexplained variability expressed as a proportional error.

## 4 Discussion

The literature presents an ongoing debate regarding differences between ALL *vs.* non-ALL patients in the V_d_ and Cl of gentamicin. Given the increased body fat composition in ALL patients (Egnell et al., 2022), some differences in central and peripheral V_d_ can be anticipated when comparing ALL to non-ALL patients. Similarly, disturbances in lean body mass and renal function associated with cytotoxic medication lead to changes in serum creatinine and Cl_cr_ (Aapro and Launay-Vacher, 2012, Mach et al., 2022), consequently affecting gentamicin clearance (Ho et al., 1995). The current analysis revealed no significant difference in Cl and/or V_d_ between ALL *vs.* non-ALL patients, an outcome that potentially contradicts expectations: as gentamicin is a hydrophilic molecule, its V_d_ is predicted to be lower in ALL patients due to higher fat content. In our study, both groups had similar characteristics in terms of age, weight, and BMI and received the same dose in mg/kg, indicating that the study results were not confounded by these factors. The only noted difference between the two groups was in non-ALL patients who had a higher Cl_cr_ compared to the ALL patients. This variation could be attributed to elevated serum creatinine and BUN, although not significant, in ALL compared to non-ALL patients secondary to receiving chemotherapeutic agents that might compromise their renal functions. This finding differs from those of Aquino et al. (Aquino et al., 2023) relating enhanced Cl_cr_ to the use of chemotherapy.

In contrast to our findings, Llanos-Paez found oncology patients to have a lower V_d_, indicating the need for lower doses in these patients compared to non-oncology patients. Several studies have also determined that ALL patients have higher levels of body fat compared to healthy matched controls. Such an increase in body fat might be attributed to the use of glucocorticoid treatment, which would increase energy intake and reduce expenditure or increase fat deposition secondary to growth hormone suppression in ALL patients (Murphy et al., 2006, Aquino et al., 2023). Regardless of the reasons behind increased body fat, V_d_ in these patients was reported to be affected. Contrariwise, our results did not reveal any difference in V_d_. This outcome might be attributed to reduced glucocorticoid treatment in the ALL patients compared to treatments applied in the non-ALL group, a factor requiring further investigation, as it was not collected for our patients.

Limitations of our study include its retrospective nature and the fact that the study relied on routine TDM data. In addition to the small sample size, several variables impacting gentamicin V_d_, such as critical illness and edema, were not available in our data set and could have influenced our results and masked the effect of ALL (Lingvall et al., 2005, Murphy et al., 2006, Murphy et al., 2010, Choi et al., 2011, Crcek et al., 2019).

In conclusion, we did not identify any statistical difference in V_d_ between ALL and non-ALL pediatric patients. According to this outcome, dosing requirements should be similar between the two groups. Future studies are needed to confirm our findings.

**Funding**

This project was funded by King Abdullah International Medical Research Center (KAIMRC, grant number NRC21R/127/04).

## CRediT authorship contribution statement

**Hisham S. Abou-Auda:** Conceptualization, Formal analysis, Investigation, Validation, Writing – original draft. **Fatimah Alotaibi:** Conceptualization, Data curation, Investigation, Validation, Writing – original draft. **Sary Alsanea:** Conceptualization, Project administration, Writing – original draft, Writing – review & editing. **Abdulrahman Alwhaibi:** Conceptualization, Data curation, Writing – original draft, Writing – review & editing. **Mohammed M. Almutairi:** Conceptualization, Data curation, Writing – original draft, Writing – review & editing. **Ziyad Alrabiah:** Methodology, Supervision, Visualization, Writing – review & editing. **Abdullah Alsultan:** Data curation, Methodology, Software, Writing – original draft. **Majed Al Jeraisy:** Conceptualization, Funding acquisition, Investigation, Project administration, Writing – review & editing.

## Declaration of competing interest

The authors declare that they have no known competing financial interests or personal relationships that could have appeared to influence the work reported in this paper.
